# Review of the genus *Metopheltes* Uchida, 1932 (Hymenoptera, Ichneumonidae) with description of a new species from Vietnam

**DOI:** 10.3897/BDJ.2.e1061

**Published:** 2014-03-05

**Authors:** Alexey Reshchikov, Kees van Achterberg

**Affiliations:** † Department of Zoology, Swedish Museum of Natural History, Stockholm, Sweden; ‡ Department of Terrestrial Zoology, Naturalis Biodiversity Center, Leiden, Netherlands

**Keywords:** Ctenopelmatinae, Perilissini, *Metopheltes*, *Metopheltes clypeoarmatus***sp. n.**, *Metopheltes chinensis* (Morley, 1913), *Metopheltes petiolaris* Uchida, 1932, Vietnam, Russian Far East

## Abstract

A new species of the genus *Metopheltes* Uchida (Hymenoptera: Ichneumonidae, Ctenopelmatinae), *Metopheltes clypeoarmatus* **sp. n.** is described from Vietnam. *Metopheltes petiolaris* Uchida, 1932 is recorded for the first time from the Russian Far East. The other previously described species are also illustrated and discussed.

## Introduction

The genus *Metopheltes* was described as a monotypic genus by Toichi Uchida in 1932, with *Metopheltes petiolaris* Uchida, 1932 from Japan as the type species (Uchida 1932). It was placed in the tribe Perilissini of the subfamily Ctenopelmatinae (Hymenoptera, Ichneumonidae) and is considered to be closely related to the genus *Opheltes* Holmgren, 1859. But twenty years before another species of this genus, *Metopheltes chinensis* (Morley, 1913) was discovered from China and placed within the genus *Opheltes* (Morley 1913). It was transferred to the genus *Metopheltes* by Henry Townes (Townes et al. 1965).

The members of the genus are rather rare as well as Ctenopelmatinae in general in the Oriental region. This species-rich subfamily includes mostly koinobiont endoparasitoids of sawfly larvae (Hymenoptera: Symphyta) and is more diverse in temperate than tropical zones as the primary host groups are relatively scarce in tropical habitats (Gauld 1997, Veijalainen et al. 2012). We know 60 species of Ctenopelmatinae recorded from the Oriental region, mostly China, while in contrast 757 species are known from the Western Palaearctic (Yu et al. 2012). On the other hand sawfly diversity in South East Asia is expected to be rather high (Wei and Niu 2009) and the history of Ctenopelmatinae research in this region is rather recent when compared with Europe.

Nothing is known about the biology of *Metopheltes*. However species of the closely related (see Diagnosis) genus *Opheltes* were reared from large-bodied sawflies of the genera *Cimbex* Olivier, 1790 and *Agenocimbex* Rohwer, 1910 (Hymenoptera, Cimbicidae) (Aubert 2000, Giraud and Laboulbène 1877, Sheng et al. 2004, Townes 1945, Townes et al. 1965, Uchida 1928, Uchida 1930, Ulbricht 1909, Zirngiebl 1961). Hosts of *Metopheltes* members could be expected within cimbicid sawflies associated with deciduous trees. Here we provide a detailed up-to-date diagnosis of the genus and a description of a new species (*Metopheltes clypeoarmatus* **sp. n.** from Vietnam) is added. *Metopheltes petiolaris* Uchida, 1932 is recorded for the first time from Russian Far East.

## Materials and methods

**Figure 1.**
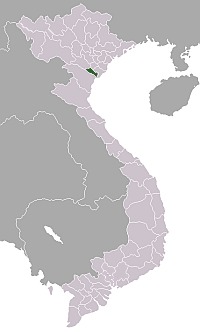
Distribution of *Metopheltes clypeoarmatus* **sp. n.**

The specimen of *Metopheltes clypeoarmatus* **sp. n**. was collected with a Malaise trap in the Cuc Phuong National Park (Vietnam) in 2000 (Fig. 1). The biotope was a small piece of disturbed lowland rainforest on limestone just inside the park and about 1 km from the headquarter buildings. The holotype of the new species is deposited in the Naturalis Biodiversity Center, Leiden (RMNH). We also studied specimens of *Metopheltes petiolaris* Uchida, 1932 from the Zoological Museum of Moscow State University (ZMUM) and the Smithsonian Institution (USNM) and *Opheltes glaucopterus* (Linnaeus 1758) from the Swedish Museum of Natural History (NHRS). The only known specimen of *Metopheltes chinensis* which is actually the type was not available for examination due to a loan policy of the British Museum of Natural History (BMNH) and we had to use photos. The morphological terminology follows Gauld (Gauld 1997). Photographs of the specimens, excluding *Metopheltes chinensis*, were taken with a Canon EOS 7D digital camera and combined using Zerene®.

## Taxon treatments

### Metopheltes

Uchida, 1932

Metopheltes Metopheltes petiolaris Uchida, 1932 – Uchida 1932

#### Diagnosis

*Metopheltes* shares several character states with *Opheltes*: the presence of a thyridium on the second tergite of the metasoma (Fig. 2c), the absence of a distinct tyloid on the basal flagellomere and a deep groove extending the full length of the mesopleuron (Fig. 3c) (Townes 1970). This mesopleural character is not unique within Perilissini and occurs also in *Priopoda impressa* Reshchikov, 2012 (Reshchikov 2012) and some Westwoodiini (Wharton et al. 2010). Unlike *Opheltes* (Fig. 4a) the second maxillary palpomere is not modified in *Metopheltes* (Fig. 2d) while the frontal carina (a character state previously found only in *Opheltes*) is present in *Metopheltes clypeoarmatus* **sp. n.** (Fig. 3b), though less developed. *Metopheltes* also differs from *Opheltes* by the shape of the propodeum in lateral view: the basal part rounded (elevated at an acute angle in *Opheltes*); its apical part comparatively elongate and the apical transverse carina not elevated (Figs 2a, 4b). The median apical ligulate process of the last visible sternite of male is notched apically and laterally (Fig. 5f). The tip of the aedeagus is bent over and ends in an adze-like blade.

### Metopheltes chinensis

(Morley, 1913)

Opheltes chinensis Morley, 1913 – Morley 1913: 135.

#### Materials

- **Type status:** Holotype. **Occurrence:** recordedBy: Fortune; individualCount: 1; sex: male; **Location:** country: China; **Event:** eventDate: 1854; **Record Level:** institutionCode: BMNH

#### Distribution

China.

#### Notes

This species is represented by a single male specimen. In the original description (Morley 1913) the type locality is not clearly mentioned, and the label data is poor, "a single male in British Museum is labelled "Northern China", but was more probably taken about Shanghai, by Mr. Fortune." (Morley 1913). However photos of the type specimen (Fig. 6) allow one to distinguish this species from closely related *Metopheltes petiolaris*. In future if more representative material of *Metopheltes* can be collected it will be possible to clarify the status of this species and provide an identification key for the group.

### Metopheltes clypeoarmatus

Reshchikov & Achterberg sp. n.

urn:lsid:zoobank.org:act:BB674DDC-E6FB-4E0D-9A6A-60CF17DB5C4A

#### Materials

- **Type status:** Holotype. **Occurrence:** recordedBy: Mai Phu Quy; individualCount: 1; sex: female; **Location:** country: Vietnam; stateProvince: Ninh Binh; verbatimLocality: Cuc Phuong N.P., near entrance, c. 225 m; **Event:** samplingProtocol: Malaise trap; eventDate: 14.iv.-1.v.2000; **Record Level:** institutionCode: RMNH

#### Description

Body length 11 mm. Antennal flagellum with 34 segments. Width to length ratio of scapus 0.5 (Fig. 3a). First flagellomere 3 times as long as wide, without distinct tyloid. Head not narrowed behind the eyes, temple rounded. Maximal length of temple 1.3 times transverse eye diameter; minimal length of temple equal to transverse eye diameter. Face as wide as longitudinal eye diameter; moderately convex, bulging; matt; densely and shallowly punctate, densely pubescent. Frontal carina between eye and antennal socket present (Fig. 3b). Clypeus flat, very slightly separated from face by a shallow impression; apical margin of clypeus moderately obtuse and serrate. Posterior ocellus separated from eye by 2.5 times maximum diameter of ocellus. Tentorial pits large (Fig. 3a). Width of malar space 0.3 times of basal width of mandible. Lower mandible tooth longer than upper one. Second maxillary palpomere not modified (Fig. 2d). Hypostomal carina joining occipital carina well above base of mandible (Fig. 2d). Occipital carina complete.

Mesosoma matt, punctate, with sparse yellowish setae. Notauli not impressed. Epicnemial carina raised at lower part of mesopleuron, not reaching anterior margin of mesopleuron, terminating dorsally in rounded transverse ridge that sharply delimits a median longitudinal furrow extending across middle of mesopleuron. Mesopleuron matt, densely and shallowly punctate, with deep groove extending full length of mesopleuron (Fig. 3c). First tibia with an apical tooth. Apical margin of mid tibia without distinct tooth similar to that on fore tibia. Posterior hind tibial spur at least 11 times longer than its maximum basal width. Tarsal claws pectinate with comparatively short teeth (Fig. 2e). Areolet of fore wing petiolate. Radius leaving pterostigma little before its middle. Second recurrent vein with two bullae. Nervulus postfurcal. Nervellus intercepted above its middle. Propodeal carinae complete, strongly raised, except basal part of dorso-median longitudinal carina; apical transverse carina curved towards metasoma; area superomedia trapezoidal and as long as wide (Fig. 3d).

Metasoma slightly shiny, smooth, sparsely pubescent. First metasomal tergite 0.4 times wider than its length (Fig. 2b); slightly prominent dorsally in profile; without shallow median longitudinal impression; only basally bordered by lateral longitudinal carinae. Second metasomal tergite with thyridium (Fig. 2c). Apical metasomal segments compressed laterally. Ovipositor straight, as long as height of last tergite, without notch and nodus apically, swollen basally (Fig. 2f).

**Figure 7.**
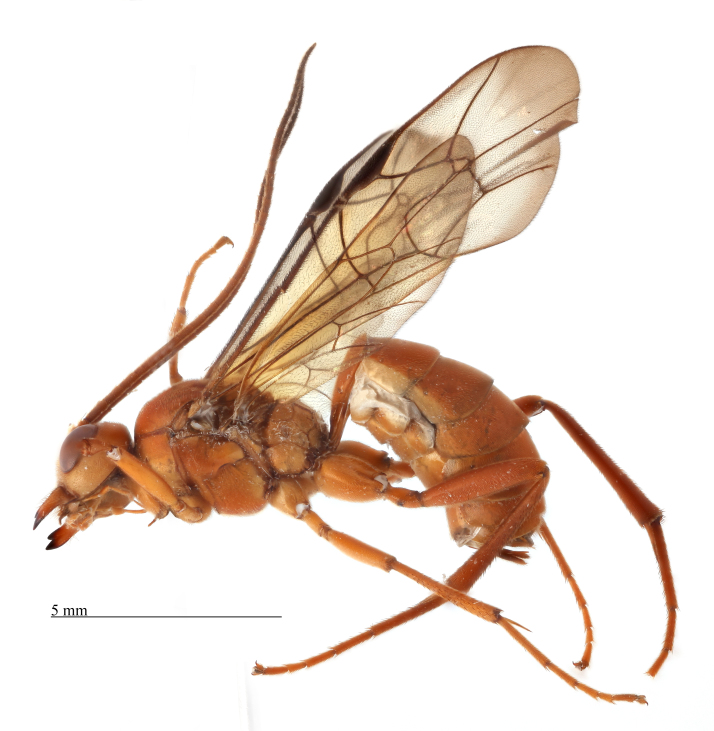
Holotype female *Metopheltes clypeoarmatus* **sp. n.**, habitus in lateral view.

Colour. Body yellowish-red (Figs 2, 3, 7). Mandible black apically (Fig. 3a). Apical part of antennal flagellum, hind tibia and tarsus reddish. Pterostigma brown. Fore wing very slightly infuscate apically.

#### Diagnosis

This species differs from other two members of *Metopheltes* by the following combination of character states: first flagellomere shorter (3.0 times as long as wide) than in *Metopheltes petiolaris* (6.0 times as long as wide) and *Metopheltes chinensis* (4.0 times as long as wide Fig. 6b clypeus); clypeus apically serrate (Fig. 3a); tentorial pits large (Fig. 3a); clypeus flat, very slightly separated from face by a shallow impression; posterior ocellus separated from eye by 2.5 times its maximum diameter (1.7 times in two other species Fig. 6a); frontal carina between eye and antennal socket present (Fig. 3b); upper hind part of mesopleuron polished and smooth (Figs 3c, 5a; punctate in *Metopheltes petiolaris*); apical margin of middle tibia without distinct tooth similar to that on fore tibia; posterior hind tibial spur at least 11.0 times longer than maximum basal width (6.0–7.0 times in *Metopheltes petiolaris*); hind femur and tibia 4.8 and 7.0 times as long as wide, respectively, (10.0 and 11.0 times in *Metopheltes petiolaris*); tarsal claws shorter and pectinate with comparatively short teeth (Figs 2e, 5d); areolet petiolate; radius leaving pterostigma only little before its middle; propodeum more precipitous (Fig. 2a) than in *Metopheltes petiolaris* (Fig. 5c), its carinae complete (Fig. 3d) and strongly raised (except basal part of dorso-median longitudinal carina; only area apicalis defined in *Metopheltes petiolaris* (Fig. 5b) and *Metopheltes chinensis* (Fig. 6c); first metasomal tergite (Fig. 2b) 0.4 times wider than long (0.6 times in *Metopheltes petiolaris* or *Metopheltes chinensis* Fig. 6d*chinensis*); ovipositor without notch and nodus apically, (ovipositor with shallow notch and weak nodus in *Metopheltes petiolaris* Figs 2f, 5e).

#### Etymology

The species epithet *clypeoarmatus* refers to the serrate apical margin of the clypeus.

#### Distribution

N. Vietnam.

### Metopheltes petiolaris

Uchida, 1932

#### Materials

- **Type status:** Other material. **Occurrence:** recordedBy: T. Fukai; individualCount: 1; sex: female; **Location:** country: Japan; stateProvince: Wakasa; **Record Level:** institutionCode: USNM
- **Type status:** Other material. **Occurrence:** recordedBy: A. Zhelokhovtsev; individualCount: 1; sex: female; **Location:** country: Russia; stateProvince: Primorsky Krai; verbatimLocality: Spassk-Dalny; **Event:** eventDate: 17.vi.1961; **Record Level:** institutionCode: ZMUM
- **Type status:** Other material. **Occurrence:** recordedBy: A. Romanov; individualCount: 1; sex: male; **Location:** country: Russia; stateProvince: Primorsky Krai; verbatimLocality: around Vladivostok; **Event:** eventDate: 28.vi.1940; **Record Level:** institutionCode: ZMUM
- **Type status:** Other material. **Occurrence:** recordedBy: A. Rasnitsyn; individualCount: 2; sex: male; **Location:** country: Russia; stateProvince: Primorsky Krai; verbatimLocality: Khasansky District, Kedrovaya Pad Nature Reserve; **Event:** eventDate: 6.vi.1962; **Record Level:** institutionCode: ZMUM

#### Diagnosis

This species differs from the other two members of *Metopheltes* by the following combination of character states: first flagellomere longer (6.0 times as long as wide) than in other species; ventrally clypeus not serrate; posterior ocellus separated from eye by 1.7 times its maximum diameter; frontal carina between eye and antennal socket absent; upper hind part of mesopleuron punctate; apical margin of middle tibia with distinct tooth similar to that on fore tibia; posterior hind tibial spur at least 6.0 times longer than maximum basal width; hind femur and tibia 10.0 and 11.0 times as long as wide, respectively; tarsal claws long and pectinate with long teeth (Fig. 5c); propodeum acclivous (Fig. 5c), not precipitous like in *Metopheltes clypeoarmatus* **sp. n.** (Fig. 2a), its carinae incomplete, only area apicalis defined (Fig. 5b); first metasomal tergite 0.6 times wider than long; ovipositor with shallow notch and weak nodus.

#### Distribution

Japan, Russian Far East (first record).

## Supplementary Material

XML Treatment for Metopheltes

XML Treatment for Metopheltes chinensis

XML Treatment for Metopheltes clypeoarmatus

XML Treatment for Metopheltes petiolaris

## Figures and Tables

**Figure 2a.**
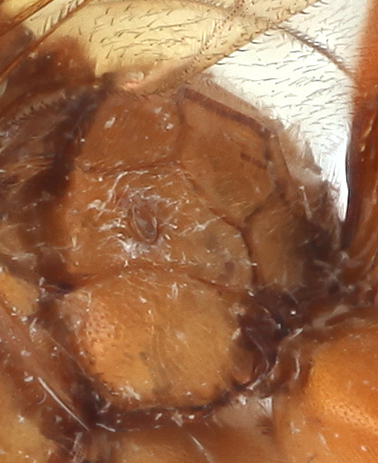
Propodeum in profile.

**Figure 2b.**
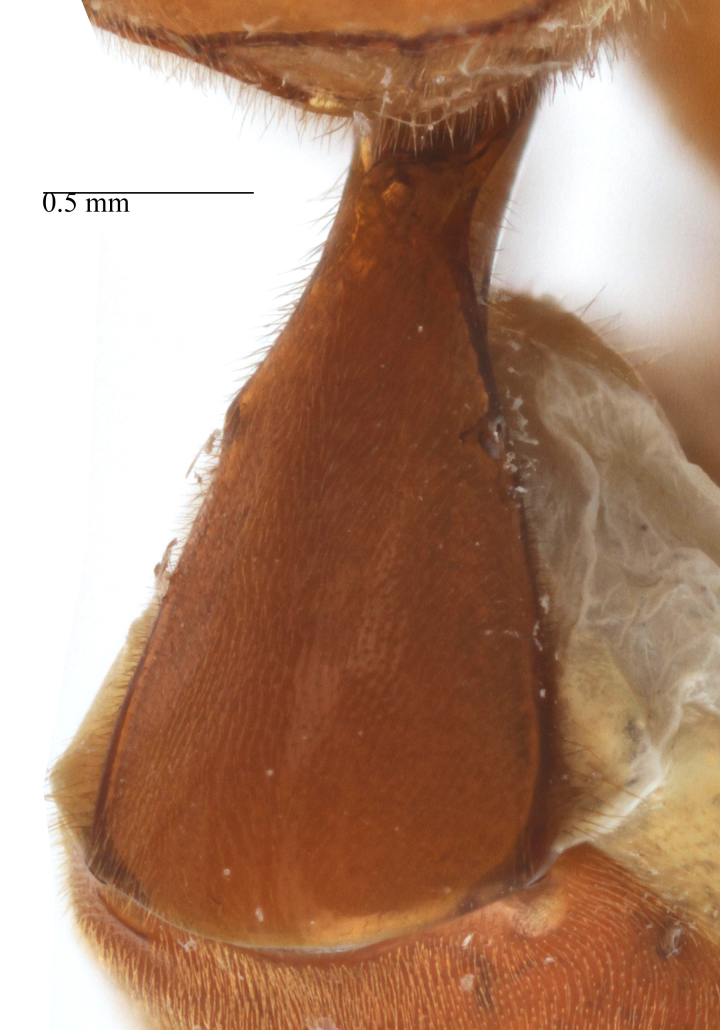
First metasomal tergite.

**Figure 2c.**
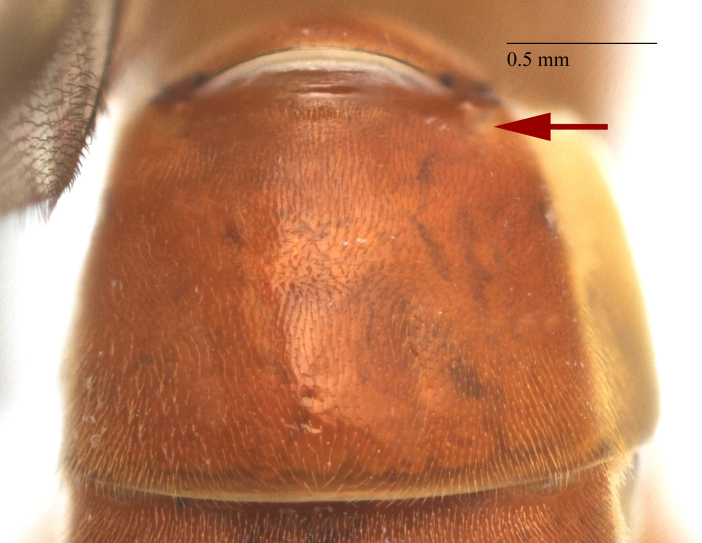
Second metasomal tergite.

**Figure 2d.**
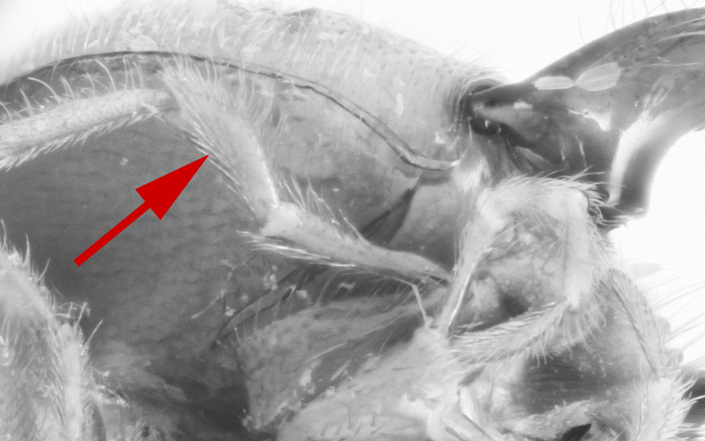
Second maxillary palpomere.

**Figure 2e.**
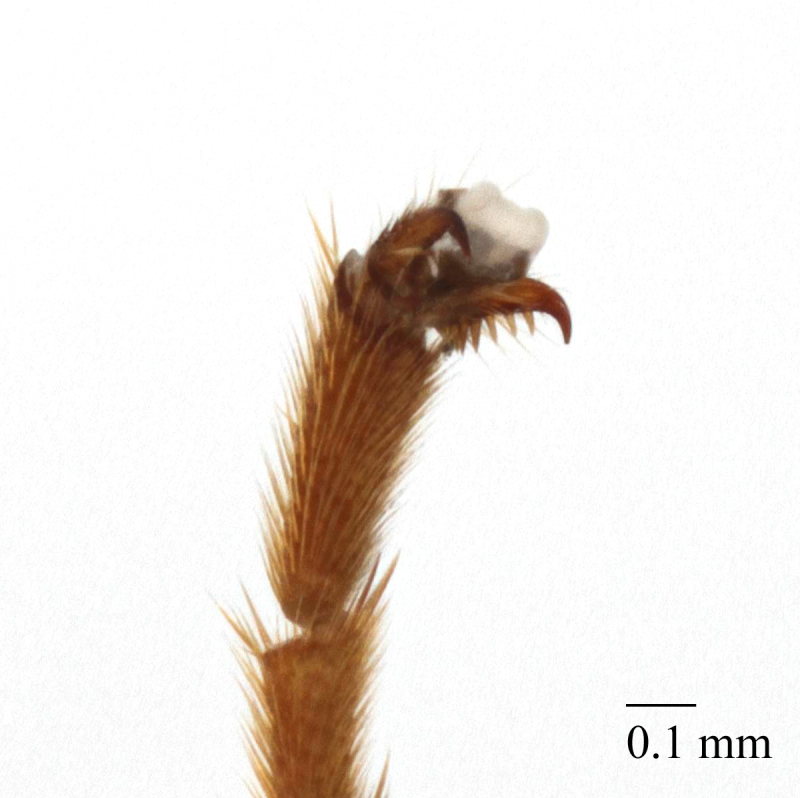
Tarsal claw.

**Figure 2f.**
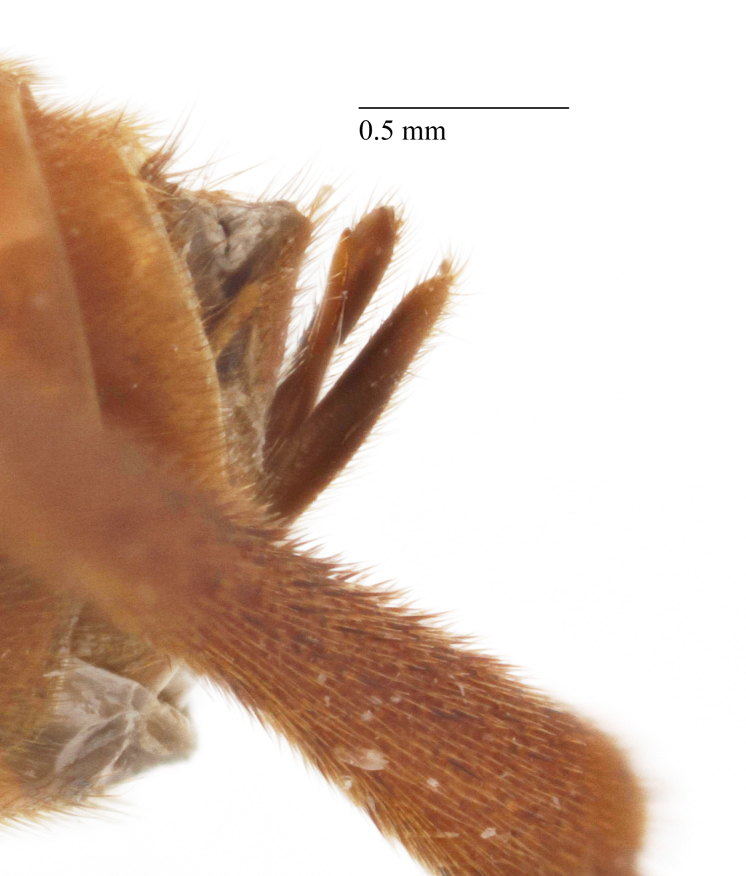
Ovipositor.

**Figure 3a.**
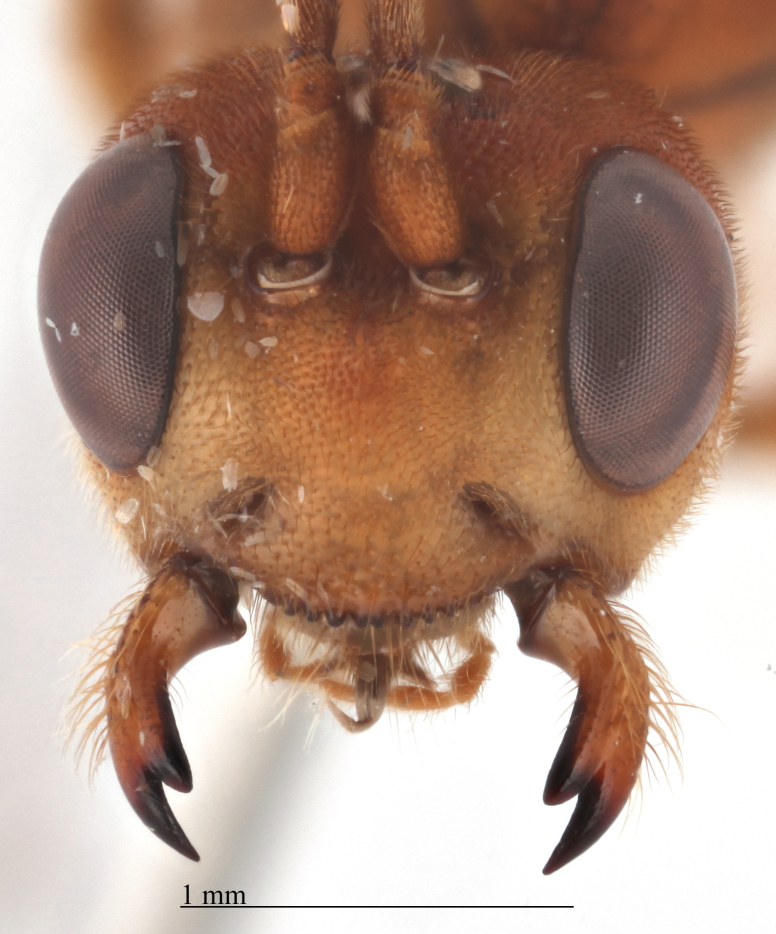
Face.

**Figure 3b.**
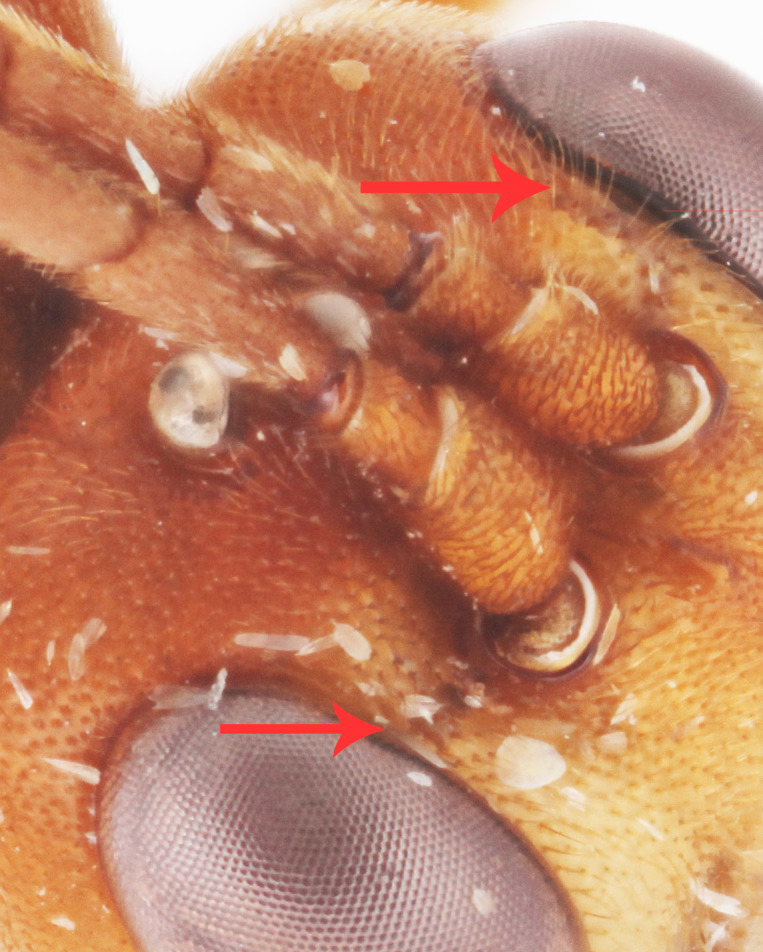
Frontal carina.

**Figure 3c.**
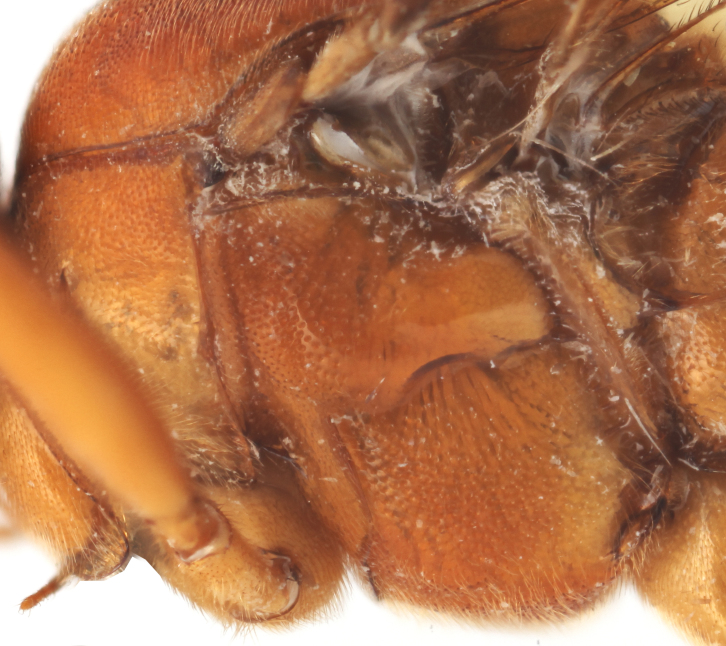
Mesopleuron.

**Figure 3d.**
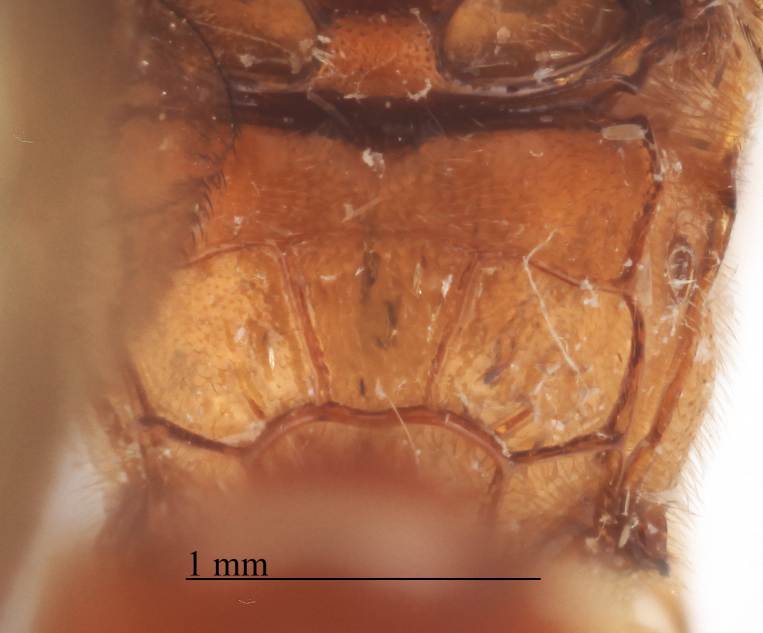
Propodeum in dorsal view.

**Figure 4a.**
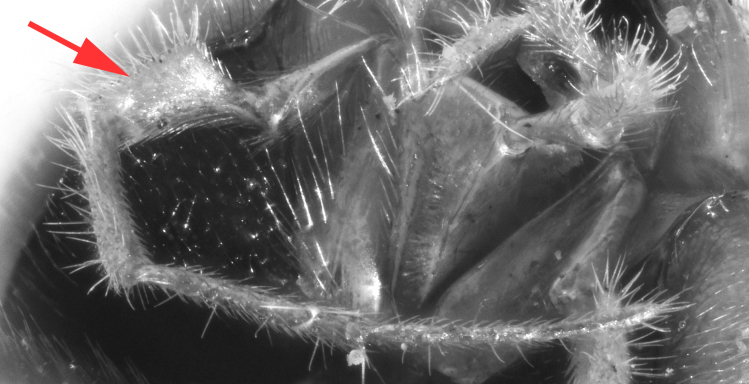
Second maxillary palpomere.

**Figure 4b.**
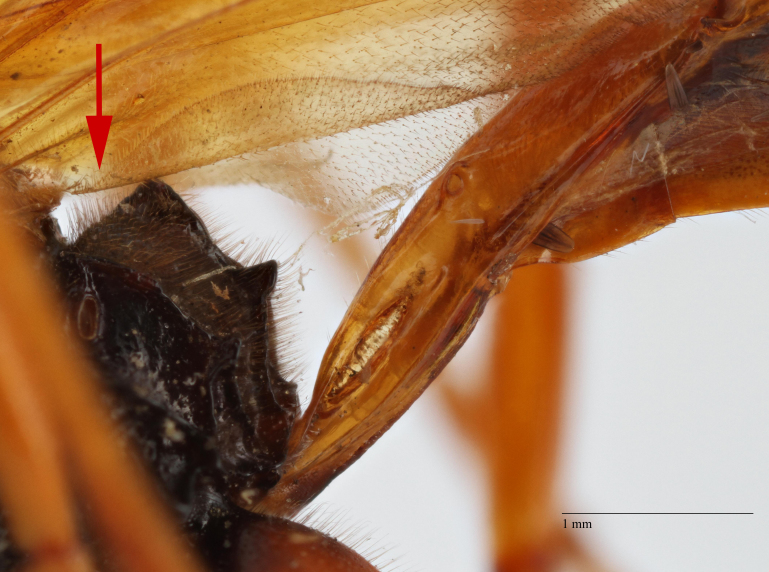
Propodeum in profile.

**Figure 5a.**
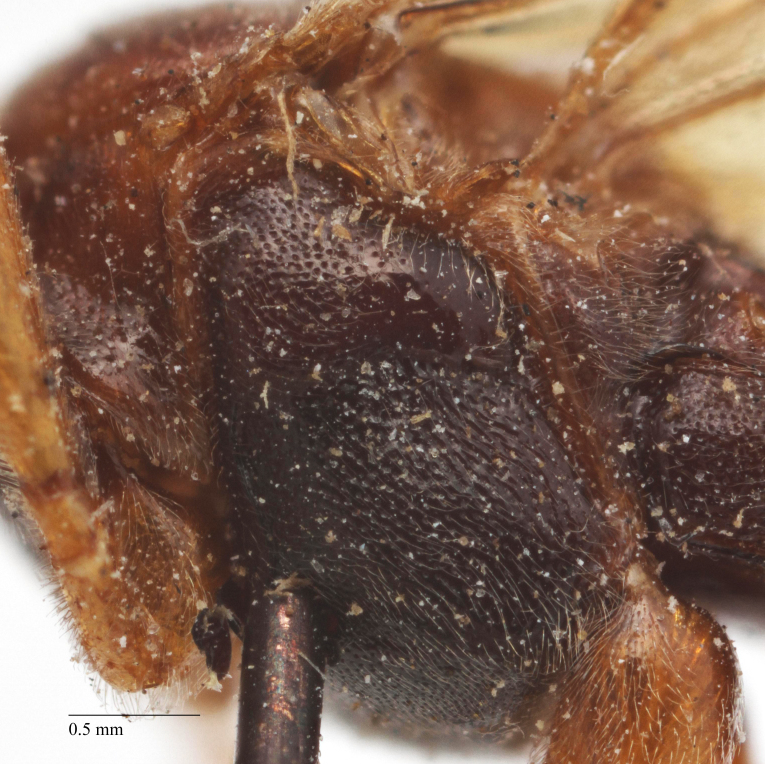
Mesopleuron, female.

**Figure 5b.**
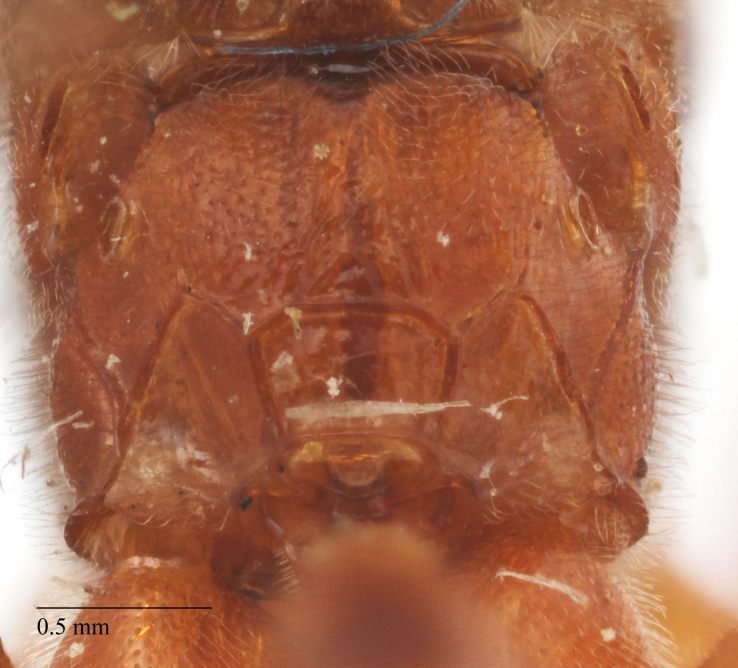
Propodeum in dorsal view, female.

**Figure 5c.**
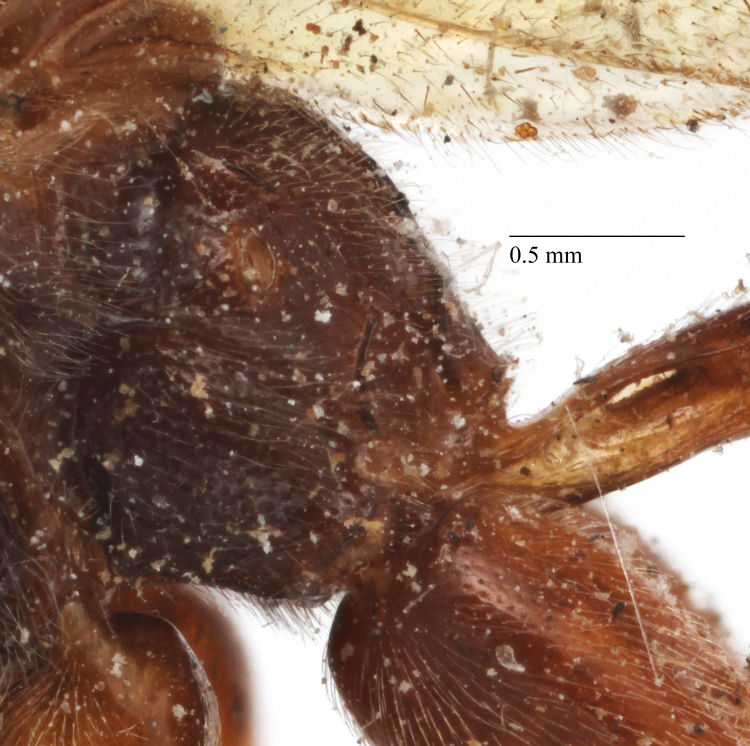
Propodeum in profile, female.

**Figure 5d.**
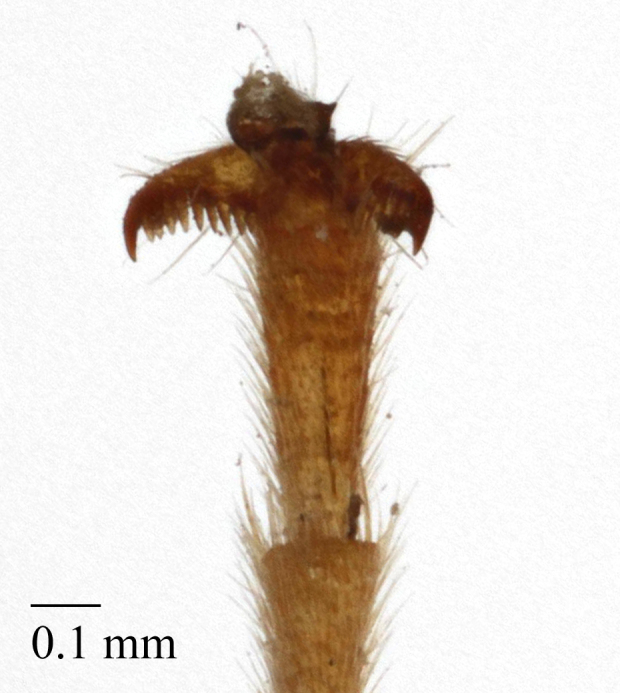
Tarsal claw, female.

**Figure 5e.**
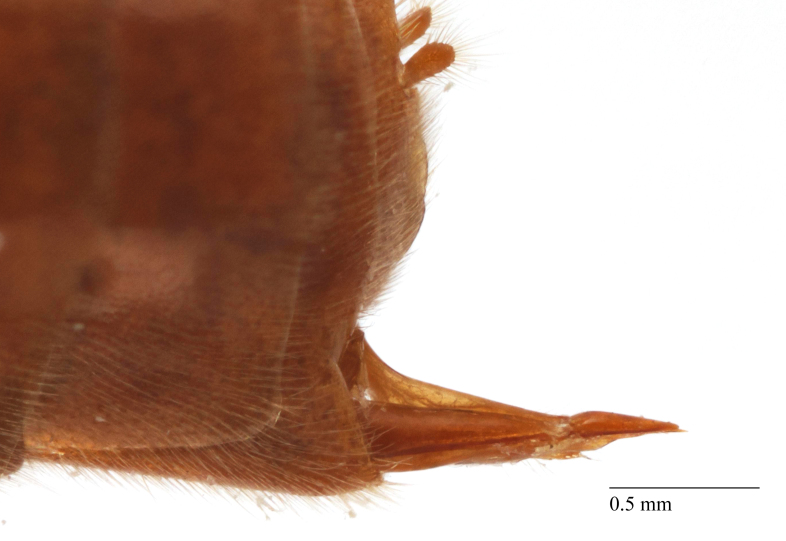
Ovipositor, female.

**Figure 5f.**
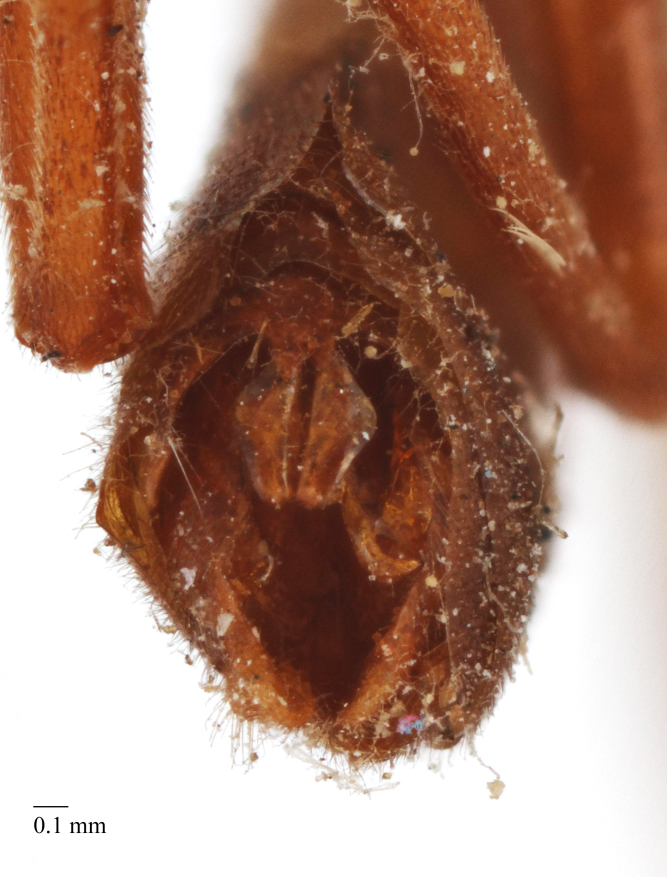
Last visible sternite, male.

**Figure 6a.**
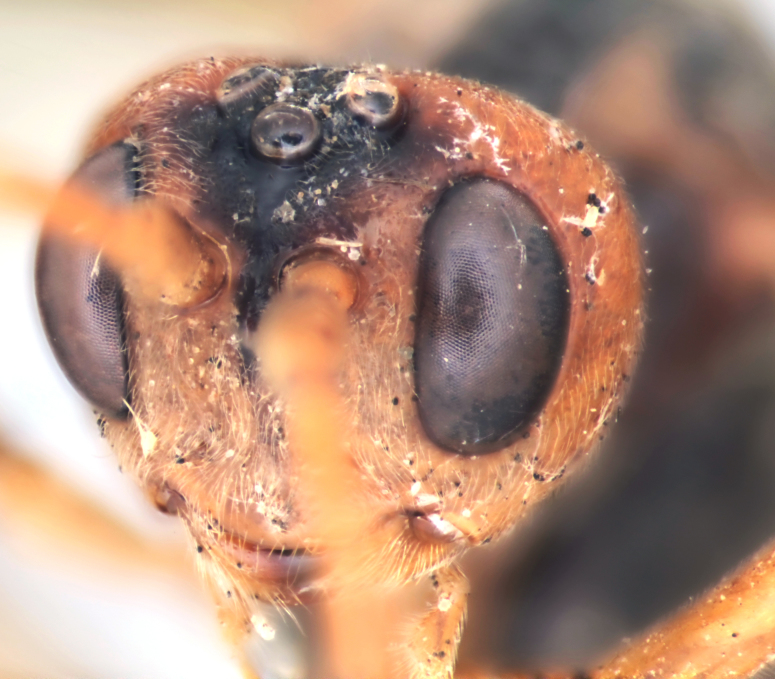
Face.

**Figure 6b.**
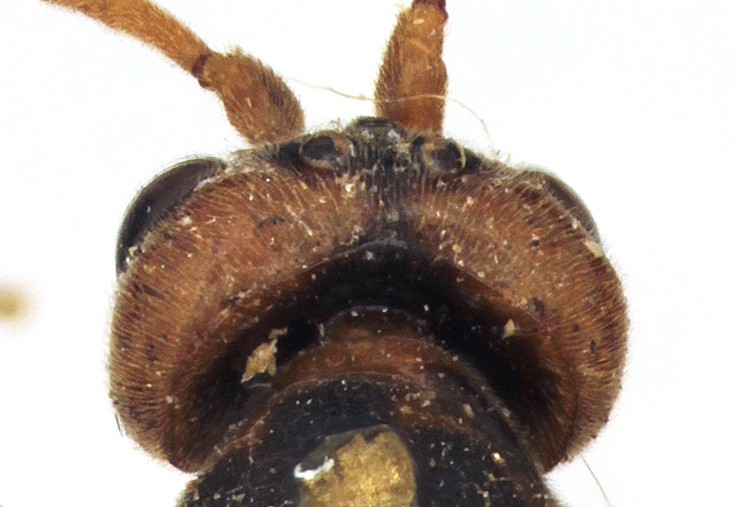
Head in dorsoposterior view.

**Figure 6c.**
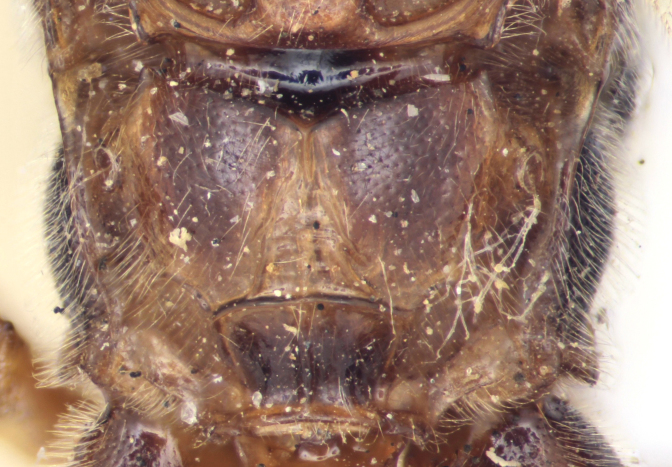
Propodeum.

**Figure 6d.**
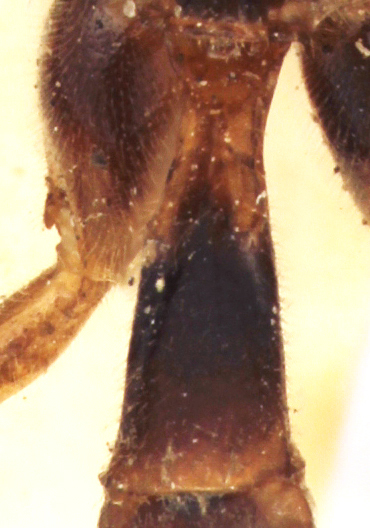
First metasomal tergite.
